# Factors affecting the quality of life of single mothers compared to married mothers

**DOI:** 10.1186/s12888-020-02586-0

**Published:** 2020-04-15

**Authors:** Ga Eun Kim, Eui-Jung Kim

**Affiliations:** Department of Psychiatry, College of Medicine, Ewha Womans University, 25, Magokdong-ro 2-gil, Gangseo-gu, Seoul, 07804 Korea

**Keywords:** Quality of life, Single parents, Single mothers

## Abstract

**Background:**

In this study, we aimed to compare the quality of life (QOL) of single mothers with that of married mothers and to identify the sociodemographic and psychological factors affecting single mothers’ QOL. We identified the factors that were similar and different between single and married mothers.

**Methods:**

We analyzed survey data obtained from 195 single mothers and 357 married mothers living in an urban community in South Korea. The QOL was assessed with the World Health Organization Quality of Life-abbreviated form (WHOQOL-BREF). All participants completed the following self-report questionnaires: the Global Assessment of Recent Stress, the Center for Epidemiologic Studies-Depression Scale, the Scale for Suicide Ideation, the Korean version of the Alcohol Use Disorder Identification Test, and the WHOQOL-BREF. These self-rating scales were used as continuous variables. Multiple linear regression analysis was performed to examine the association of quality of life with the sociodemographic and psychological factors for single and married mothers.

**Results:**

Single mothers showed lower QOL than married mothers. Older age, high income and education level, and professional job status were positively correlated with the QOL of single mothers. Residential instability, higher stress levels, depressive symptoms, suicidal ideation, and alcohol-related problems were negatively associated with the QOL of single mothers. Multiple regression analysis suggested that residential instability (public rental housing: β = − 10.779, *p* <  0.001; Jeonse rental housing: β = − 0.324, *p* = 0.01) and alcohol-related problems (β = − 0.522, *p* <  0.001) were independent factors affecting lower QOL, whereas professional job status (β = 8.452, *p* = 0.037) was independently associated with higher QOL in single mothers. However, these factors were not associated with the QOL of married mothers. Higher education level was independently associated with higher QOL in both groups (β = 3.149, *p* <  0.033 in single mothers, β = 12.052, *p* <  0.001 in married mothers).

**Conclusions:**

Higher education level was associated with higher QOL in both groups. Unlike in married mothers, type of residence and occupation (related to the economic level) had a significant impact on QOL in single mothers. Alcohol-related problems were significantly correlated to QOL in single mothers compared to married mothers.

## Background

The number of single-parent families in Korea increased from 18,119,000 in 2012 to 19,524,000 in 2017. The proportion of single-parent families among all households increased from 9.9% in 2012 to 10.9% in 2017 [1]. The crude divorce rate (per 1000 people) more than tripled from 1.1 in 1990 to 3.4 in 2003 in Korea [2]. This sharp increase in divorce rates may have been partly due to the economic crisis in Korea in the late 1990s [3]. The economic crisis led to employment instability and aggravated economic polarization [4]. Economic difficulties can lead to increased dissatisfaction with married life due to emotional distress and marital conflicts and may thus increase the possibility of family conflict and family breakup [3]. Since the crisis, income inequality has led to increased rates of depression and suicide [5]. The increasing divorce and suicide rates eventually led to an increase in the proportion of single-parent families.

Single parents, particularly single mothers, are a socially and economically vulnerable group that is at risk for various physical and mental health problems. For instance, single mothers have poor physical and mental health status relative to parents living as couples [6, 7]. According to the 2015 Single Parent Household Survey in Korea, the predominant type of single-parent household (43.7%) consisted of a mother and one or more children [8]. The average monthly income for single-parent households was 1.869 million won per month, which was less than half of the average national monthly household income, and the average income for households headed by single mothers was even lower [8]. The survey also reported that vulnerable single parents with low education and income levels were more likely to experience depressive symptoms and report worse subjective health conditions [8]. A recent review suggests that single mothers have lower health status levels than married mothers and that financial strain and lack of social support were associated with the differences in health status [6]. Another study found that unemployment, poverty, and lower education were associated with poor health in single mothers [7]. Moreover, single parents report experiencing more prejudice against them [9]. The single-parent status of families is mostly due to divorce, after which single parents often feel that they have failed in their lives and may struggle with perceived negative opinions of people around them [9].

Traditional indicators, such as the frequency and severity of disease, are insufficient for defining health status; increasingly, health assessments have been including estimates of well-being and quality of life (QOL) [10]. The World Health Organization (WHO) defines the QOL as “individuals’ perceptions of their position in life in the context of the culture and values systems in which they live and in relation to their goals, expectations, standards and concerns” [10]. The WHO developed a QOL measurement tool in response to the need for international assessments of the QOL and committed to the introduction of a holistic approach to health and healthcare [10]. Assessing the QOL of single parents can assess not only their health status but also perceived health status in relation to their cultural, social, and environmental backgrounds [10].

Single mothers reported lower life satisfaction and lower health status than married mothers [6, 11]. Conversely, one study reported that single mothers’ life satisfaction was higher in countries with supportive family policies and higher levels of gender equality [12]. Wang et al. reported that major depression was only significantly more prevalent in single mothers aged 25–50 years and that economic hardships and weak social support did not significantly affect the rate of major depressive syndrome in this population [13]. The results of studies evaluating life satisfaction and health status in single mothers are inconsistent. In addition, existing studies have used different definitions for life satisfaction and health status. To our knowledge, there is no study comparing the QOL of single mothers and married mothers in Asia using reliable assessment tools.

In this study, we aimed to evaluate the QOL of single mothers, who account for the highest percentage of single parents, compared with that of married mothers using the World Health Organization Quality of Life-abbreviated form (WHOQOL-BREF). In addition, we intended to identify the sociodemographic status and psychological factors that affect QOL in this group when compared to married mothers in order to identify any factors that specifically affect single mothers’ QOL.

## Methods

### Subjects

The study subjects included 195 single mothers in Yangcheon-gu who completed a mental health survey about single-parent families. This survey was conducted between June 7 and June 24, 2011, as part of a community survey project conducted by the Yangcheon-gu Mental Health and Welfare Center. It was aimed at creating a healthcare plan targeting parents and children in single-parent households. Nineteen dongs in Yangcheon-gu were classified into three district strata according to similar local characteristics. A total of 497 single-parent households were selected by stratified random sampling. Thirty surveyors visited their households and explained the survey purpose and the instructions for completing the questionnaire. Written consent was collected, and the self-report questionnaire was distributed. The survey was not conducted if the survey subjects were absent. The completed questionnaires were collected on a second visit. The response rate was 59%, and a total of 291 single-parent households participated. For this study, we excluded single-parent families that were headed by single fathers, grandparents, and never-married mothers (mothers who have children but were never married); survey data from the remaining 195 single-mother families (divorced mothers and widows) were included in the analysis. We believe that many families did not want to be identified as single-parent households even though the survey was anonymous.

Married mothers were selected from the same city’s 2009 community survey project for mental healthcare conducted by the Yangcheon-gu Mental Health and Welfare Center. A total of 1000 community members were selected using stratified random sampling. They were visited at their homes by the surveyors for face-to-face interviews. We used the same questionnaire as used when surveying the single mother group. After excluding men and unmarried women, we surveyed the remaining 357 married mothers. The study was approved by Institutional Review Board of the Ewha University Medical Center.

### Assessment method

Self-report questionnaires were used to obtain information about the participants’ sociodemographic characteristics, including age, education, occupation, monthly average income, type of housing, and household ownership. The QOL and psychological problems that may affect it, such as recent stress, depressive symptoms, suicidal ideation, and alcohol use problems, were evaluated using a self-rating scale. QOL scores and all psychological variables were analyzed as continuous variables.

The QOL was assessed using the WHOQOL-BREF, which was translated into Korean according to a formal standardization process [14]. The WHOQOL-BREF is derived from the comprehensive WHOQOL assessment and was developed as a brief but accurate survey instrument that is more practical for certain clinical and other applications [15]. It consists of 26 items which are rated on a five-point scale and are grouped into four domains: physical health domain, psychological domain, social relationship domain, and environmental domain [15]. The physical health domain has 7 questions regarding pain and discomfort, energy and fatigue, sleep and rest, mobility, activities of daily living, dependency on medication or treatment, and work capacity [14]. The psychological domain has 6 questions regarding positive feelings, thinking, self-esteem, body image and appearance, negative feelings, and spirituality [14]. The social relationships domain has 3 questions regarding personal relationships, practical social support, and sexual activity [14]. The environmental domain has 8 questions regarding physical safety and security, home environment, financial resources, health and social care, opportunities for acquiring new information and skills, participation and opportunities for recreation/leisure, physical environment, and transport (see appendix) [14]. The Cronbach α-value for the total score was 0.882 in single mothers and 0.909 in married mothers.

Depressive symptoms were evaluated with the Center for Epidemiologic Studies-Depression Scale (CES-D), which consists of 20 items rated on a 0–3 point scale [16]. Thus, the total score range is 0–60 points, and higher scores indicate higher levels of depressive symptoms. The reliability and validity of this scale have been confirmed by Cho and Kim [17]. The Cronbach α-values for the CES-D score were 0.876 in single mothers and 0.878 in married mothers. The Global Assessment of Recent Stress (GARS) was used to assess recent stress [18]. The Korean translation of the GARS by Koh and Park was used in this study [19]. This scale consists of eight items designed to be self-scored on a 0–9 point scale, with higher scores indicating higher levels of perceived stress [19]. The Cronbach α-value for the GARS score was 0.914 in single mothers and 0.907 in married mothers. The Beck Scale for Suicide Ideation (SSI-Beck) was used to assess suicidal ideation [20]. This scale consists of 19 items designed to be self-scored on a 0–2 point scale, with higher scores indicating higher levels of suicidal ideation. We used the Korean version that was translated by Shin [21]. The Cronbach α-value for the SSI-Beck score was 0.863 in single mothers and 0.919 in married mothers. The Alcohol Use Disorder Identification Test (AUDIT-K) was used to evaluate alcohol-related problems. This scale was developed by the WHO as a screening instrument for hazardous and harmful consumption of alcohol [22]. We used the Korean version that was translated by Lee et al. [23]. This scale consists of 10 items designed to be self-scored on a 0–4 point scale. Higher scores indicate a higher likelihood of hazardous and harmful use of alcohol as well as possible alcohol dependence [24]. The Cronbach α-value for the AUDIT-K score was 0.923 in single mothers and 0.832 in married mothers.

### Analysis

The Chi-square test was used for group comparisons that included categorical variables (education level, occupation, monthly income, and housing type). Continuous variables (age and psychological variables) were examined using the t-test. Pearson correlation analysis was performed to examine the correlations between the continuous variables (age and psychological variables) in single and married mothers. Univariate linear regression analysis was performed to determine the variables that were associated with the QOL of single and married mothers. The variables associated with the QOL that were identified in the univariate linear regression were selected as independent variables in multiple linear regression to assess how they independently affected the QOL. Age, education level, occupation, monthly income, housing type, GARS score, the CES-D score, the SSI score, and the AUDIT-K score were entered as independent variables in single mothers. Age, education level, occupation, monthly income, the GARS score, the CES-D score, the SSI score, and the AUDIT-K score were entered as independent variables in married mothers. Stepwise regression analyses were performed to identify predictor variables from among the socio-demographic, psychological variables. Dummy variables were used when independent variables (education level, occupation, monthly income, and housing type) were categorical. Single and married mothers were analyzed separately. All statistical analyses were conducted with the Statistical Package for the Social Sciences (SPSS) version 20.0 (IBM Corp., Armonk, NY), and the significance level was set at *p* <  0.05.

## Results

### Characteristics of the study subjects

There was no significant difference in the average age of single mothers (42.7 ± 7.8 years) and married mothers (43.4 ± 8.8 years). Single mothers had relatively lower education levels (*p* = 0.006); 67.2% of single mothers were high school graduates and 26.2% were college graduates, whereas 53.2% of married mothers were high school graduates and 36.1% were college graduates. Furthermore, 86.7% single mothers worked outside the home, whereas only 33.9% married mothers worked outside the home (*p* <  0.001). Regarding single-parent households, 57.9% had an average monthly income of less than two million KRW, and 36.4% had an average monthly income of two million to five million KRW. Meanwhile, in the control group (two-parent households), 3.1% of households had an average monthly income of less than two million KRW, and 86.8% had an average monthly income of two million to five million KRW. These results indicate that single mothers more often have low income compared to married mothers (*p* <  0.001). More than half (59%) of single-parent households lived in Jeonse rental housing (requires security deposit), 16.4% lived in monthly rental housing, and 12.8% lived in public rental housing. In contrast, more than half of two-parent households (67.5%) were homeowners. A significantly smaller proportion of single mothers lived in owned houses compared to married mothers (*p* <  0.001) (Table 1).

**Table 1:** Participants’ sociodemographic and psychological characteristics

	Single mothers (*N* = 195) N (%)	Married mothers (*N* = 357) N (%)	*p*
Sociodemographic characteristics			
Age^a^	42.7 ± 7.8	43.4 ± 8.8	0.347
Education level			0.006^*^
Middle school graduate	13 (6.7)	38 (10.6)	
High school graduate	131 (67.2)	190 (53.2)	
Above college	52 (26.2)	129 (36.1)	
Occupation			< 0.001^*^
Housewife or student	26 (13.3)	236 (66.1)	
Production	124 (63.6)	89 (24.9)	
Indoor job	40 (20.5)	25 (7.0)	
Profession	5 (2.6)	7 (2.0)	
Monthly income (10 thousand won)			< 0.001^*^
< 200	113 (57.9)	11 (3.1)	
200–499	71 (36.4)	310 (86.8)	
≥ 500	11 (5.6)	36 (10.1)	
Residence			< 0.001^*^
Owner	23 (11.8)	241 (67.5)	
Jeonse rental housing	115 (59.0)	104 (29.1)	
Monthly rental housing	32 (16.4)	7 (2.0)	
Public rental housing	25 (12.8)	5 (1.4)	
Psychological variables			
WHOQOL-BREF score^a^	72.0 ± 10.6	86.9 ± 13.1	< 0.001^*^
CES-D score^a^	20.9 ± 8.3	12.5 ± 7.8	< 0.001^*^
GARS score^a^	33.1 ± 12.0	19.7 ± 10.4	< 0.001^*^
SSI-Beck score^a^	3.6 ± 5.1	2.8 ± 5.0	0.067
AUDIT-K score^a^	7.1 ± 7.1	5.8 ± 4.2	0.023^*^

^*^p < 0.05

^a^ Mean ± SD (Standard deviation)

WHOQOL-BREF: World Health Organization Quality of Life-Abbreviated form (WHOQOL-BREF)

*CES-D* Center for Epidemiologic Studies Depression Scale, *GARS* Global Assessment of Recent Stress, *SSI-Beck* Beck Scale for Suicide Ideation, *AUDIT-K* Korean version of the Alcohol Use Disorder Identification Test

Single mothers had lower QOL scores than married mothers (72.0 ± 10.6 vs. 86.9 ± 13.1) and were also more likely to have depressive symptoms (20.9 ± 8.3 vs. 12.5 ± 7.8), higher levels of recent stress (33.1 ± 12.0 vs. 19.7 ± 10.4), and alcohol-related problems (7.1 ± 7.1 vs. 5.8 ± 4.2) compared to married mothers (Table 1).

### Factors influencing quality of life in single mothers and married mothers

Table 2 shows the correlations among age, psychological factors, and QOL scores in single mothers and married mothers. Increased age was significantly positively correlated with the QOL in the single mother group (*r* = 0.208, *p* < 0.01), whereas age was negatively correlated with the QOL in the married mother group (*r* = − 0.150, *p* < 0.01). Depressive symptoms, stress level, suicidal ideation, and alcohol-related problems were significantly negatively correlated with the QOL of both single and married mothers.

**Table 2:** Correlations analysis among age, psychological variables, and QOL in single mothers and married mothers

	1.Age	2.QOL	3.Stress	4.Depressive symptoms	5.Suicidal ideation	6.Alcohol problems
**Single mothers **(*N* = 195)						
1	1	0.208^*^	−0.241^*^	−0.311^*^	−0.11	−0.226^*^
2		1	−0.254^*^	− 0.232^*^	− 0.210^*^	−0.363^*^
3			1	0.506^*^	0.006	0.051
4				1	0.219^*^	0.251^*^
5					1	0.057
**Married mothers** (*N* = 357)						
1	1	−0.150^*^	0.046	−0.056	0.019	0.052
2		1	−0.498^*^	−0.367^*^	−0.409^*^	−0.297^*^
3			1	0.582^*^	0.340^*^	0.217^*^
4				1	0.485^*^	0.195^*^
5					1	0.338^*^

^*^p < 0.01

Table 3 shows the results of the univariate linear regression analysis evaluating the factors related to the QOL. All of the demographic variables except for age and menial job occupation showed similar associations in both single and married mother groups. All psychological variables were negatively correlated with the QOL in both groups, and only the magnitude of the association differed between groups. Single mothers who had professional occupations (compared to those who were housewives or students) and were college graduates (compared to middle school graduates) had significantly higher QOL. Higher monthly household income was associated with higher QOL in single mothers. Compared to house ownership, non-house ownership was associated with lower QOL in single mothers. Single mothers who had higher scores for stress, depression, suicidal ideation, and alcohol-related problems were more likely to have lower QOL. In the married mother group, college graduates had significantly higher QOL than middle school graduates.

**Table 3:** Association of QOL with the sociodemographic and psychological factors by univariate linear regression analyses

	Single mothers (*N* = 195)			Married mothers (*N* = 357)		
	β	S.E.	*p*	β	S.E.	*p*
**Age**	0.284	0.096	0.004^*^	−0.221	0.078	0.005^*^
**Education level**						
Middle school graduate^a^						
High school graduate	2.624	3.035	0.388	1.2	2.245	0.593
Above college	7.477	3.243	0.022^*^	8.266	2.332	< 0.001^*^
**Occupation**						
Housewife or student^a^						
Production	3.283	2.249	0.146	−3.616	1.617	0.026^*^
Indoor job	3.204	2.626	0.224	0.819	2.734	0.756
Profession	16.954	5.091	0.001^*^	6.339	4.985	0.204
**Monthly Income (10 thousand won)**						
< 200^a^						
200–499	3.087	1.542	0.047^*^	8.209	4	0.041^*^
≥ 500	13.785	3.216	< 0.001^*^	7.159	4.491	0.112
**Residence**						
Owner^a^						
Jeonse rental housing	−8.496	2.24	< 0.001^*^	−1.017	1.538	0.509
Monthly rental housing	−7.74	2.68	0.004^*^	−4.63	5.028	0.358
Public Rental housing	−17.282	2.833	< 0.001^*^	−1.144	5.9258	0.847
**GARS score**	−0.226	0.062	< 0.001^*^	−0.624	0.058	< 0.001^*^
**CES**-**D score**	−0.297	0.09	0.001^*^	−0.62	0.083	< 0.001^*^
**SSI score**	−0.44	0.148	0.003^*^	−1.061	0.126	< 0.001^*^
**AUDIT**-**K score**	−0.541	0.1	< 0.001^*^	−0.996	0.226	< 0.001^*^

^*^p < 0.05

^a^reference group

*S.E.* Standard error

*GARS* Global Assessment of Recent Stress, *CES-D* Center for Epidemiologic Studies Depression Scale

SSI-Beck: Beck Scale for Suicide Ideation, AUDIT-K: Korean version of the Alcohol Use Disorder Identification Test

Married mothers who had menial jobs or were employed in the service industry were more likely to have lower QOL than housewives in the same group. Married mothers with a monthly income of two million to five million KRW had significantly higher QOL than those with a monthly income of less than two million KRW. Similar to single mothers, married mothers who had higher scores for stress, depression, suicidal ideation, or alcohol-related problems were more likely to have lower QOL.

Table 4 shows independent factors affecting the QOL with multiple linear regression. The variables identified as factors affecting the QOL in the univariate linear regression were selected as independent variables in multiple linear regression. For single mothers, professional status and college graduation were independent factors influencing high QOL. Alcohol-related problems and living in public rental housing were also independent factors influencing low QOL in this group. Among married mothers, high education level was an independent factor associated with high QOL. Stress and suicidal ideation were found to be independent factors associated with lower QOL risk factors.

**Table 4:** Association of QOL with the sociodemographic and psychological factors by multiple linear regression analyses

		β	S.E.	*p*	Adjusted R^2^
**Single mothers**					0.332
AUDIT-K score		− 0.522	0.09	< 0.001^*^	
Residence^a^	Jeonse rental housing	−0.324	0.125	0.01^*^	
	Public rental housing	−10.779	2.166	< 0.001^*^	
Occupation^b^	Profession	8.452	4.024	0.037^*^	
Education level^c^	Above college	3.149	1.462	0.033^*^	
**Married mothers**					0.493
GARS score		−0.693	0.072	< 0.001^*^	
Education level^c^	High school graduate	6.122	2.921	0.037 ^*^	
	Above college	12.052	3.007	< 0.001^*^	
SSI score		−0.591	0.132	< 0.001^*^	

^*^p < 0.05

Reference group: ^a^ owner, ^b^ housewife or student, ^c^ middle school graduate

*S.E.* Standard error

AUDIT-K: Korean version of the Alcohol Use Disorder Identification Test

*GARS* Global Assessment of Recent Stress, *SSI-Beck* Beck Scale for Suicide Ideation,

## Discussion

This study examined the sociodemographic characteristics and psychological factors affecting the QOL of single mothers. The mean WHOQOL score was significantly lower in the single mother group (72.0 points) than in the married mother group (86.9 points). Single mothers were more likely to have higher stress levels, depressive symptoms, and alcohol-related problems than married mothers. These results suggest that single mothers have poorer mental health and poorer QOL than married mothers. In a previous study, single mothers were more likely to experience higher levels of chronic stress and depression than married mothers, and they reported lower levels of perceived social support and less frequent contact with friends and family members [25]. Crosier et al. reported that single mothers had poorer mental health than married mothers and that economic difficulties and lack of social support were the strongest predictors for a lower mental health status [26]. Single mothers typically have poorer health than married mothers and they are more socioeconomically disadvantaged [27]. Therefore, it is expected that the comprehensively-assessed QOL scores in all domains—physical, psychological, social, and environmental—are lower in this group.

We found that single mothers had relatively lower educational levels and lower average monthly household income than married mothers. Moreover, they were more likely to work outside the home compared to married mothers. In a previous study, it was reported that single mothers were less educated and poorer than married mothers [28]. Single mothers reported that they were likely to have jobs that did not provide enough income to meet their needs [28]. The 2015 Single Parent Household Survey found that 87.4% of the survey respondents were employed at the time of the survey. One-year before becoming a single-parent family, 56.3% of the respondents were employed; after becoming single parents, the percentage of employed persons was 60.8%, indicating a higher rate of employment after becoming a single parent. Single mothers may be forced to get a job because they are responsible for the family economy. However, to accommodate their childcare schedule, they may seek hourly or temporary work, with the majority engaged in service or menial jobs.

The residential status of single mothers was less stable than that of married mothers. The frequency of home ownership was remarkably low in single mothers than in married mothers. Housing type in Korea is a reflection of their economic status. Public rental housing is a system in which the government leases houses to low-income residents. Jeonse is a type of housing deposit that is returned to the renter when the lease expires. In general, people who live in public rental housing and monthly rental housing tend to a have lower economic status than those living in other residence types. The 2015 Single Parents Household Survey reported that 21.2% of single parents owned their own home and that housing stability was low in this population [8]. In particular, the home ownership rate was low in single parents aged under 30 years and in those who had lower educational levels, had preschool-age children, were temporary workers or unemployed, and had low income.

The univariate linear regression analyses showed similar associations between all variables for both single and married mothers except for age and menial occupation.

Stress, depressive symptoms, suicidal ideation, and alcohol-related problems were negatively correlated with the QOL in both groups. Previous studies have also reported that severe depression and suicidal ideation correlated with lower health-related QOL [29, 30]. Another study found that the QOL was low in patients with alcohol use disorders, particularly in the aspects of mental and social functioning [31]. In addition, stress plays an important role in determining the QOL [32].

Higher education level was associated with higher QOL in both groups. Higher education level was an independent factor for improved QOL after adjusting for other factors affecting the QOL. Well-educated people typically have lower levels of psychological and physical distress because they are more likely to be employed, engage in non-alienating work, and have more economic resources [33]. Well-educated people also have a greater sense of control over their lives and have more opportunities for employment, higher incomes, and more social and psychological resources [33]. A previous study reported that married women who are highly educated experience more life satisfaction and have more positive emotions [34]. Single mothers with higher educational attainment showed higher levels of family-life satisfaction, active participation in family activities, and strong family cohesion during crises [35]. In addition, the higher was the education level, the higher was the perception and level of social support [35]. Single mothers with low income are burdened with childcare costs, perceive that their income is insufficient, and experience more difficulties in raising and educating their children [35].

Age was positively correlated with the QOL of single mothers; however, age was negatively correlated with the QOL of married mothers. A previous study of Korean married women also reported that as age increases, psychological well-being, subjective well-being, and health-related QOL decrease [36, 37]. A possible explanation is the different situations of single and married mothers; single mothers are responsible for the household economy as well as childcare. Older single mothers may have had more time to adapt after becoming single mothers and are more likely to have older children; thus, they may experience less burden and stress of parenting than younger single mothers with younger children [35]. In the 2015 Single Parent Households Survey, younger age in single parents and younger age of the youngest child were both associated with increased constraints related to job and career opportunities. In addition, the rate of receiving low-income single-parent public assistance was the highest among single parents younger than 30 years and in those who had the youngest children (preschool- and primary-school-aged children) [8].

Single-mother professionals have a higher QOL than single-mother homemakers. Even after adjusting for other factors that affect the QOL, professional employment remained a factor associated with higher QOL. Single mothers have responsibilities to provide financial support, and having confidence in their ability to do so is important for their self-esteem [38]. Financial difficulty is a major problem for single women, except for those who are professional workers [39]. Professional workers are mostly satisfied with their income level, which relates to higher life satisfaction [39]. In addition, engaging in a profession provides more than just economic rewards; it also assures a sense of accomplishment, connection to a broader world, sense of belonging, less loneliness, and greater satisfaction with life [39].

However, among married mothers, those in menial jobs were more likely to have lower QOL than those who were homemakers. When married women are engaged in menial jobs to earn a living, it may be more difficult for them to feel pride or a sense of accomplishment, and consequently, their QOL is likely to be lower.

In both groups, the higher was the monthly income, the higher was the QOL; however, this association was more prominent in the single mother group than in the married mother group. Married women seem to have other factors affecting their QOL when their income is above a certain level; in contrast, economic level is a major factor affecting the QOL of single mothers. If the housing type is not home ownership, the QOL tends to decline in single mothers. In multiple linear regression, the residence type independently affected the QOL differently in single and married mothers. Housing instability reflects single mothers’ economic difficulties. In the 2015 survey, the most important support reported by single-parent families was “cash support such as cost of living and parenting expenses,” followed by “housing support such as facilities and rental housing,” suggesting that there was much desire for economic support [8]. A previous study found that financial hardship was the primary factor affecting poor mental health in single mothers [26]. Another study has shown that economic hardship, the difficulty of child-rearing, and social prejudice were the biggest problems faced by single mothers, while child-rearing and social prejudice were the biggest problems for single fathers [40]. Taken together, economic hardship is one of the most difficult problems for single-parent families headed by single mothers, and consequently, economic difficulty is a major factor affecting the QOL.

Among the assessed psychological problems, alcohol use problems negatively affected the QOL even after adjusting for other factors affecting the QOL of single mothers but not of married mothers. Women with alcohol dependency experience more loss and frustration associated with the death of a family member, divorce, domestic violence, and serious marital conflicts when compared to men [41]. Alcohol-dependent women were more likely to live alone due to widowhood or divorce, which were high-risk factors for alcohol dependency [41]. Unlike men whose initial drinking motivators were interpersonal, women with alcohol-related problems reportedly begin drinking to escape from the negative emotions associated with life problems [42]. Women’s drinking is associated with psychological and emotional factors. Women with alcohol-related problems feel guilt and shame, and they drink alcohol alone and become isolated because of social prejudice and lack of support; thus, detection and treatment are delayed [42]. Single mothers may use alcohol to escape from negative feelings, such as depression and loneliness, due to marital conflict, divorce, and bereavement. Previous studies have reported that single mothers experience stigma and lack of social support [25, 26]. Lack of social support and social prejudice are factors that exacerbate negative emotions and alcohol problems, thereby lowering the QOL.

The limitations of this study are as follows: First, we obtained data from self-report questionnaires; thus, objective criteria were not evaluated, and there is a possibility that the participants exaggerated or underreported their problems. Second, the subjects were sampled from a single community; regional characteristics cannot be disregarded, and generalizing the findings to all single mothers requires caution. Third, the causal relationships between variables were difficult to evaluate due to the cross-sectional study design. Fourth, we compared single mothers based on results of a survey conducted in 2011 to married mothers based on a survey conducted in 2009. The two groups are not directly comparable since the surveys were performed at different time periods.

Nonetheless, one strength of this study is that QOL was assessed using standardized measurement tools. In addition, the sociodemographic and psychological factors influencing the QOL were comprehensively evaluated in both single mothers and married mothers in two-parent households. Notably, the type of residence and occupation, which are both related to socioeconomic level, influenced the QOL more remarkably in single mothers than in married mothers. In addition, having alcohol-related problems was an important factor associated with the QOL in the single mother group. Single mothers need social support and resources to prevent and mitigate alcohol-related problems.

## Conclusions

Single mothers have lower QOL than married mothers. We identified how several sociodemographic and psychological factors differently affect QOL in single mothers and married mothers. Higher education level was associated with higher QOL in both groups. The type of residence and occupation (both are related to economic level) had a significant impact on the QOL in the single mother group but not in the married mother group. This finding indicates that economic difficulties more strongly affected the QOL of single mothers than of married mothers. In addition, the presence of alcohol-related problems was significantly correlated with decreased QOL in single mothers when compared with married mothers. Evaluating the QOL of single mothers and identifying the factors influencing it can help develop the policies and services required to improve their health status. It is necessary to pay attention to economic hardships and alcohol-related problems when attempting to improve the QOL of single mothers. Policies targeting economic stability as well as early detection of alcohol-related problems and related interventions are needed to assist this population and improve their QOL.

## Data Availability

The datasets used during the current study are available from the corresponding author on reasonable request.

## Appendix

### Structure of the WHOQOL-BREF

**Table:**

WHOQOL-BREF domains	Number of questions
Overall Quality of Life and General Health	2
Domain 1 - Physical health domain	
Pain and discomfort	1
Energy and fatigue	1
Sleep and rest	1
Mobility	1
Activity	1
Medication	1
Work capacity	1
Domain 2 - Psychological domain	
Positive feelings	1
Thinking	1
Self esteem	1
Bodily image	1
Negative feelings	1
Spirituality	1
Domain 3 - Social relationships domain	
Personal relationships	1
Social support	1
Sexual activity	1
Domain 4 - Environmental domain	
Safety and security	1
Home environment	1
Financial resources	1
Health and social care	1
Opportunities for acquiring new information and skills	1
Participation and opportunities for recreation/leisure	1
Physical environment	1
Transport	1

## Abbreviations

AUDIT-K: Korean version of the alcohol use disorder identification test
CES-D: Center for epidemiologic studies depression scale
GARS: Global assessment of recent stress
QOL: Quality of life
SD: Standard deviation
S.E.: Standard error
SSI-Beck: Beck scale for suicide ideation
WHOQOL-BREF: World Health Organization Quality of Life-Abbreviated form (WHOQOL-BREF)
